# Water-Modulated Synthesis of MIL-68(Al) and MIL-53(Al) in Protonic and Aprotic Solvents

**DOI:** 10.1021/acsomega.6c08862

**Published:** 2026-10-06

**Authors:** Motomu Sakai

**Affiliations:** † Department of Industrial Chemistry, Tokyo University of Science, 6-3-1 Nijuku, Katsushika-ku, Tokyo 125-8585, Japan; ‡ Department of Applied Chemistry, Waseda University, 513 Wasedatsurumaki-cho, Shinjuku-ku, Tokyo 162-0041, Japan

## Abstract

Al-based MOFs, MIL-53­(Al) and MIL-68­(Al), were synthesized in organic solvents (isopropyl alcohol, tetrahydrofuran, methanol, and *N*,*N*-dimethylformamide) by adding a small amount of water. The influence of water content (0–20 mol % relative to organic solvent) on crystallization behavior was studied. Pure MIL-53­(Al) and MIL-68­(Al) were obtained in water-containing organic solvents. With the addition of water, the number of crystals decreased and particle size increased. In addition, the water-modulated MOFs exhibited high crystallinity, as determined by XRD, and large BET surface areas. Water in organic solvents (both protonic and aprotic) plays a role similar to that of monocarboxylic acids in typical modulation techniques, such as the suppression of nucleation frequencies by the competitive coordination of water and ligands with metal ions. The results suggest that, in addition to its typical role, water plays another role in aprotic solvents by promoting crystal growth, possibly through enhanced ligand deprotonation.

## Graphical abstract

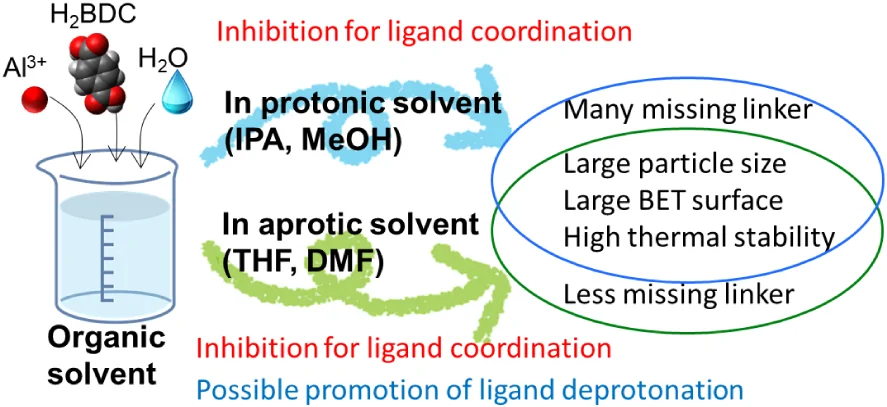

## 1 Introduction

Metal–organic frameworks (MOFs) are promising porous materials following porous carbons, silica and zeolites. MOFs have been widely studied as adsorbents for gas purification and storage because their pore structures and adsorption properties can be controlled by changing the metal–ligand combination.^1–3^ MOFs have also been widely investigated as catalysts and catalyst precursors.^4,5^ For these various applications, structural properties such as crystallinity, particle size, porosity, and framework stability are important factors affecting material performance. In addition, the toxicity, environmental compatibility, and economics of metal ions and organic ligands are also important factors in choosing MOFs. Therefore, Al-based MOFs such as CAU-1­(Al), MIL-53­(Al), MIL-68­(Al), and MIL-96­(Al) have been widely studied.^6–9^

The crystallinity, morphology, and particle size of crystalline porous materials affect their adsorption performance (adsorption–desorption rate, and adsorption capacity)^10–12^ and catalytic performance.^13,14^ Miyahara et al. investigated the effect of particle size of ZIF-8 on the diffusion and adsorption properties of molecules in both experimental and simulation studies.^11,12^ The morphology and particle size also strongly influenced the fluidity of the powder and the ease of recovery by filtration. Therefore, the development of a synthesis method that can control these factors for each process is desirable. For example, small particles are preferred for fluidized beds, whereas large particles are suitable as adsorbents in the liquid phase.^15,16^ In catalytic applications, particle size and morphology can also influence mass transfer by changing the diffusion path length within MOF crystals.

Over the past decade, modulation methods have been developed to control MOF crystallization behavior. Metal–ligand bond formation in MOF crystallization is governed by competition among ligand coordination, solvent coordination, and ligand deprotonation. To control MOF nucleation and crystal growth, additives called “modulator”, which coordinate with the metal source and compete with the original ligands, are used. Monocarboxylic acids and inorganic acids are often used as modulators. Kitagawa et al. controlled the crystal size and pore volume by adding dodecanoic acid as a modulator during the synthesis of Cu-BTC.^17^ They pointed out that the modulator suppressed the nucleation frequency, resulting in a small number of large Cu-BTC particles. Morris et al. reported the control of the crystallization behavior of UiO-66 using various carboxylic acids (formic acid, acetic acid, and trifluoroacetic acid) as modulators.^18^ Canossa and coworkers successfully synthesized Al-MOFs (MIL-53, CAU-10, MIL-69) using oxalic acid.^19^ Oxalic acid acts as a modulator by forming heteroleptic and homoleptic complexes with Al^3+^ in water. Steinert et al. studied the effect of modulators added during MIL-160 synthesis on the pore volume and surface area.^20^

I recently reported the water-modulated synthesis of CAU-1­(Al)-NH_2_.^21^ In this case, highly crystalline CAU-1­(Al)-NH_2_ was obtained in methanol by adding a small amount of water (1–3 wt %). CAU-1­(Al)-NH_2_ prepared with the addition of water had a larger particle size, a greater micropore volume, and a higher ratio of missing linkers than CAU-1­(Al)-NH_2_ synthesized in pure methanol, suggesting that water acted as a modulator for the crystallization of CAU-1­(Al)-NH_2_. Conventional modulation methods often require relatively large amounts of organic modulators, which increase the synthesis cost. In addition, residual modulator molecules would remain in MOFs after synthesis and influence their adsorption properties. Therefore, a simple modulation strategy that uses only water is attractive from both the economic and practical viewpoints. Water is inexpensive and environmentally benign compared with conventional organic modulators. In addition, water can be readily removed during activation, minimizing concerns regarding residual modulator molecules. This water modulation method has the potential to provide a general strategy for Al-based MOF synthesis.

However, the principles and general versatility of this water-modulation method remain unclear. In particular, the role of a small amount of water may depend on the nature of the organic solvent. I hypothesized that water could suppress nucleation through competitive coordination with metal ions, as observed in conventional modulation, whereas in aprotic solvents water might additionally influence linker deprotonation and thereby promote crystal growth. To examine these hypotheses and the general applicability of water modulation, MIL-53­(Al) and MIL-68­(Al) were synthesized in protic and aprotic solvents (methanol, isopropyl alcohol, *N*,*N*-dimethylformamide, and tetrahydrofuran) with different water contents (0–20 mol %). The effects of water content and solvent identity on the crystallization behavior and resulting properties of the Al-MOFs were systematically investigated.

## 2 Experimental Section

### 2.1 Reagents

The following reagents were used for MOF synthesis: AlCl_3_·6H_2_O (Sigma-Aldrich, 99%), Al­(NO_3_)_3_·9H_2_O (FUJIFILM Wako Chemical, 99.9%), terephthalic acid (H_2_BDC, FUJIFILM Wako Chemical, >95%), *N*,*N*-dimethylformamide (DMF, Kanto Chemical, >99.5%), isopropyl alcohol (IPA, Kanto Chemical, >99.7%), methanol (MeOH, FUJIFILM Wako Chemical, 99.8%), and tetrahydrofuran (THF, FUJIFILM Wako Chemical, >99.5%). A molecular sieve (zeolite 4A) was added to DMF, IPA, MeOH, and THF for drying. The organic solvents were dried to minimize the influence of residual water and to quantitatively control the amount of water intentionally added to the synthesis solution. All other reagents were used as received.

### 2.2 Preparation of MOFs

Two types of Al-based MOFs composed of terephthalic acid (MIL-68 (Al) and MIL-53­(Al)) were prepared by solvothermal synthesis. Table 1 lists the synthesis conditions.

**Table 1:** Synthesis Conditions of MIL-68­(Al) and MIL-53­(Al)

Sample name	Reagent	Solvent	Solvothermal conditions
MIL-68(Al)I-0	H_2_BDC, AlCl_3_·6H_2_O	IPA w/o H_2_O addition	348 K, 72 h
MIL-68(Al)I-4	H_2_BDC, AlCl_3_·6H_2_O	IPA with 4 mol % H_2_O	348 K, 72 h
MIL-68(Al)I-12	H_2_BDC, AlCl_3_·6H_2_O	IPA with 12 mol % H_2_O	348 K, 72 h
MIL-68(Al)I-20	H_2_BDC, AlCl_3_·6H_2_O	IPA with 20 mol % H_2_O	348 K, 72 h
MIL-68(Al)T-0	H_2_BDC, Al(NO_3_)_3_·9H_2_O	THF w/o H_2_O addition	333 K, 72 h
MIL-68(Al)T-4	H_2_BDC, Al(NO_3_)_3_·9H_2_O	THF with 4 mol % H_2_O	333 K, 72 h
MIL-68(Al)T-12	H_2_BDC, Al(NO_3_)_3_·9H_2_O	THF with 12 mol % H_2_O	333 K, 72 h
MIL-68(Al)T-20	H_2_BDC, Al(NO_3_)_3_·9H_2_O	THF with 20 mol % H_2_O	333 K, 72 h
MIL-53(Al)M-0	H_2_BDC, AlCl_3_·6H_2_O	MeOH w/o H_2_O addition	423 K, 5 h
MIL-53(Al)M-4	H_2_BDC, AlCl_3_·6H_2_O	MeOH with 4 mol % H_2_O	423 K, 5 h
MIL-53(Al)M-12	H_2_BDC, AlCl_3_·6H_2_O	MeOH with 12 mol % H_2_O	423 K, 5 h
MIL-53(Al)M-20	H_2_BDC, AlCl_3_·6H_2_O	MeOH with 20 mol % H_2_O	423 K, 5 h
MIL-53(Al)D-0	H_2_BDC, AlCl_3_·6H_2_O	DMF w/o H_2_O addition	423 K, 20 h
MIL-53(Al)D-4	H_2_BDC, AlCl_3_·6H_2_O	DMF with 4 mol % H_2_O	423 K, 20 h
MIL-53(Al)D-12	H_2_BDC, AlCl_3_·6H_2_O	DMF with 12 mol % H_2_O	423 K, 20 h
MIL-53(Al)D-20	H_2_BDC, AlCl_3_·6H_2_O	DMF with 20 mol % H_2_O	423 K, 20 h

A given amount of water (0, 4, 12, or 20 mol % relative to the organic solvent) was added to the organic solvents, and then terephthalic acid was added into the solvent. After the metal source was dissolved in a mixture of the solvent and terephthalic acid, the synthesis solution was solvothermally treated in a PTFE-lined autoclave. In each preparation, the amounts of metal source, organic ligand, and solvent were 4.00, 4.00, and 600 mmol, respectively. Thus, molar ratio of metal:ligand:organic solvent:water was 1:1:150:6 when 4 mol % water was added. For comparison, each MOF was prepared in a pure organic solvent without added water. After the solvothermal treatment, the autoclave was quenched with flowing tap water for 30 min. The synthesized crystals were then recovered by centrifugation. The collected powder was dispersed in 30 mL of DMF and stirred at room temperature for 10 min, followed by centrifugation. This DMF washing procedure was repeated twice to remove residual unreacted organic species, including terephthalic acid. Subsequently, the powder was immersed in 30 mL of methanol at room temperature overnight to replace DMF with a more volatile solvent. After recovery by centrifugation, the powder was dried at 423 K in air for at least 12 h. Crystallization temperatures and period were slightly modified from those reported in the literature: MIL-68­(Al)­I,^22^ MIL-68­(Al)­T,^22^ MIL-53­(Al)­M,^23^ and MIL-53­(Al)­D.^24^

The terminal letters (D, I, M, and T) indicate the solvents (DMF, IPA, MeOH, and THF), respectively. Numbers after the letters represent the water content of the solvent. For example, MIL-68­(Al) synthesized in IPA with 4 mol % water was denoted as MIL-68­(Al)­I-4.

### 2.3 Microscopic Observation

Prepared MOFs were observed by FE-SEM (S-4800, Hitachi High-Tech Corp.) to evaluate the morphology and particle size distribution. The accelerating voltage and working distance are 1 kV and 15 mm, respectively.

### 2.4 Thermogravimetric Analysis

To evaluate the missing-linker ratio, thermogravimetric analysis (TG) was conducted according to the literature.^25,26^ An 8.0-mg MOF sample was heated in flowing air at 5 K min^–1^. The missing-linker ratio was calculated as follows. The structural formula of MIL-68­(Al) and MIL-53­(Al) is Al­(OH)­C_8_H_4_O_4_ (formula weight: 208.10). Thermal decomposition of MIL-68­(Al) and MIL-53­(Al) yields one-half equivalent of Al_2_O_3_ (molar mass = 101.96 g mol^–1^) per formula unit. Thus, if there are no missing linkers, the weight loss upon combustion would be 75.5%. As the missing-linker ratio increased, combustion mass loss decreased. The missing-linker ratio (%), *M*_*L*_, is calculated from the mass of the dried MOFs at 673 K, *W*_673_, and the mass of the residue at 1173 K, *W*_1173_. Notably, *W*_673_ represents the weight of the completely dried MOFs in which the preadsorbed molecules are desorbed.

1 $$M_{L}\left(\%\right)=\left(1-\left(\frac{W_{1173}-W_{673}}{0.755}\right)\right)\times 100$$

### 2.5 Adsorption Measurement

N_2_ adsorption isotherms at 77 K were measured using a volumetric adsorption analyzer (3-Flex, Micromeritics) and the BET surface area of each sample was calculated from the isotherms. Before measurement, the samples were heated at 473 K for 3 h under vacuum to remove preadsorbed molecules.

## 3 Results

### 3.1 Yield and Crystal Phase

Figure 1 shows the XRD patterns of the 16 powders listed in Table 1. In the XRD results, only the diffraction paterns derived from the desired crystal phase were observed for MIL-68­(Al)­I, MIL-68­(Al)­T, MIL-53­(Al)­M, and MIL-53­(Al)­D. The intensities of the diffraction peaks increased with the addition of water. This tendency was observed in MIL-68­(Al)­I, MIL-68­(Al)­T, and MIL-53­(Al)­M. For MIL-53­(Al)­D, the peak intensity increased slightly.

**Figure 1:**
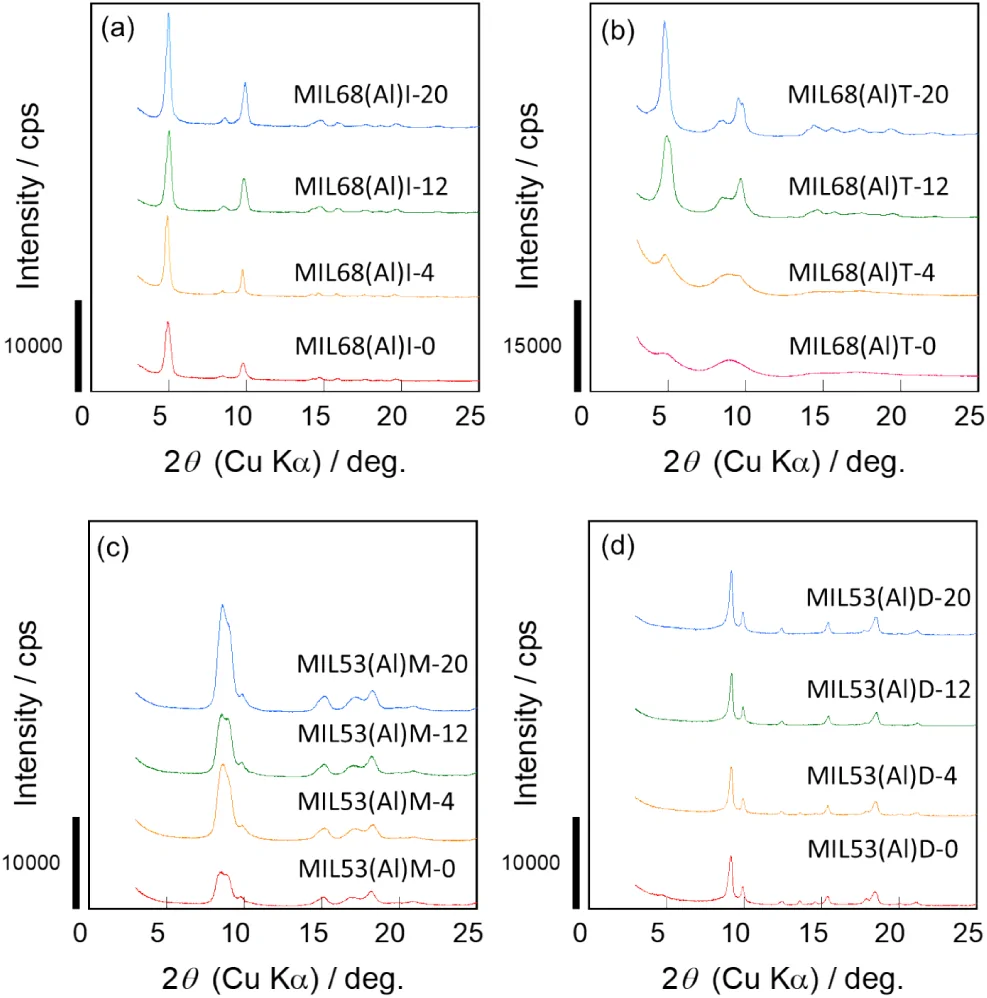
XRD patterns of (a) MIL-68­(Al)­I, (b) MIL-68­(Al)­T, (c) MIL-53­(Al)­M, and (d) MIL-53­(Al)­D.

Figure 2 shows product yields calculated from the recovered solid masses. The product yield of MIL-68­(Al)I synthesized in IPA decreased with increasing water content in solvent, from 56.9% in IPA without water to 33.1% in IPA with 20 mol % water. Similarly, the MIL-53­(Al)­M yield decreased from 60.2 to 48.9% after adding 20 mol % water. For MIL-68­(Al)­T and MIL-53­(Al)­D, yields increased with an appropriate amount of water and decreased with excess water. For example, the yields of MIL68­(Al)­T-0, MIL68­(Al)­T-4, MIL68­(Al)­T-12, and MIL68­(Al)­T-20 were 82.3, 87.5, 82.8, and 46.2%, respectively.

**Figure 2:**
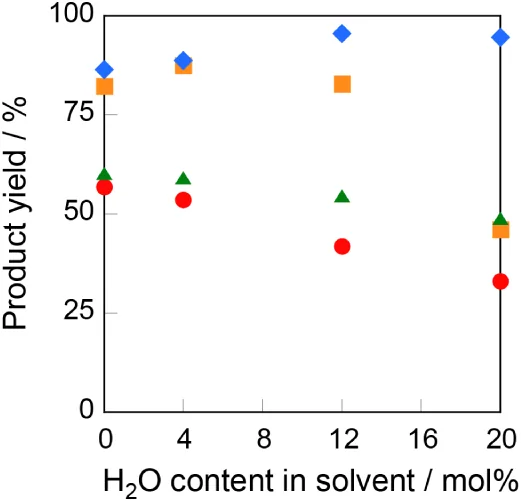
Product yield of MOFs as a function of water content in organic solvents: ◦MIL-68­(Al)­I, □MIL-68­(Al)­T, △MIL-53­(Al)­M, ◊MIL-53­(Al)­D.

### 3.2 Morphology and Particle Size Evaluated through Microscopic Observation

Figure 3 shows typical FE-SEM images and particle size distributions of the synthesized MOFs. MIL-68­(Al)I had a plate-like shape with a length of 50–500 nm. MIL-68­(Al)­T had a completely different morphology from that of MIL-68­(Al)­I, with a spherical shape of several micrometers. Both MIL-53­(Al)­M and MIL-53­(Al)­D were plate-like crystals several hundred nanometers in size.

**Figure 3:**
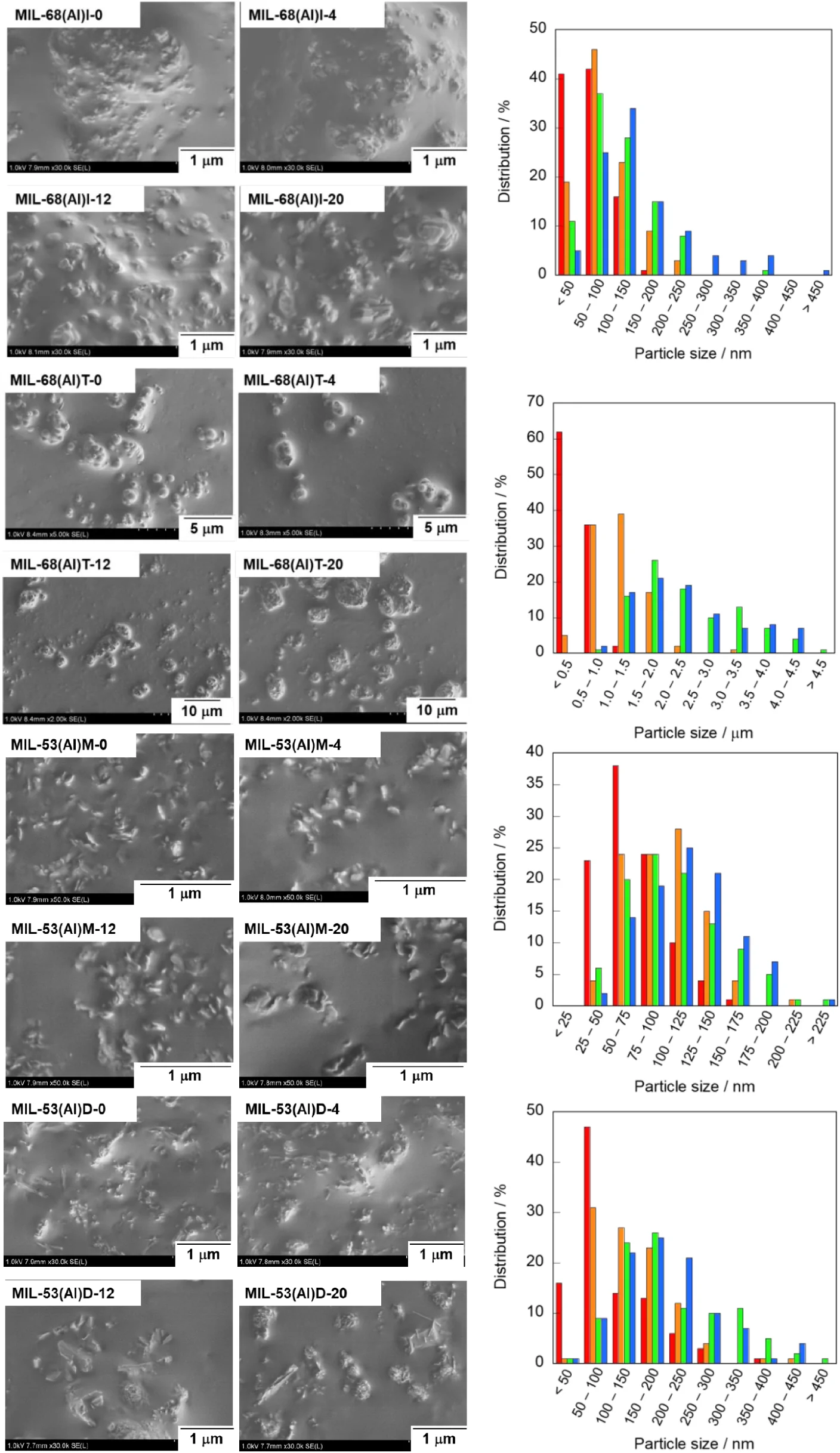
Typical FE-SEM images of prepared MOFs and particle size distributions. Red, orange, green, and blue bars show 0, 4, 12, and 20 mol % water, respectively.

The particle size distributions were evaluated by measuring the longest dimension of 100 particles under each condition. The median particle sizes are listed in Table 2. In all the MOFs, the particle size tended to increase with added water. For instance, the median particle sizes of MIL-68­(Al)­I and MIL-68­(Al)­T increased from 60 to 130 nm and 440 to 2280 nm, respectively, with the addition of water. The standard deviations also increased with the addition of water; for example, the standard deviations were 34 and 93 nm for MIL-68­(Al)­I, and 206 and 1630 nm for MIL-68­(Al)­T without and with 20 mol % water, respectively. As particle size increased, the crystal morphology also changed. The addition of water made the facets more clearly visible. Although it is difficult to quantitatively discuss crystallinity based on microscopic observations, this change in crystal morphology is consistent with the improvement in crystallinity evaluated by XRD measurements.

**Table 2:** Characteristics of MOFs Prepared^a^

Sample name	Yield/%	Median particle size/nm	Missing-linker ratio/%	BET surface area/m^2^ g^–1^	Ref
MIL-68(Al)I-0	56.9	59.9	–0.65	1108	This study
MIL-68(Al)I-4	53.6	70.1	1.95	1112	This study
MIL-68(Al)I-12	41.9	104	9.15	1307	This study
MIL-68(Al)I-20	33.1	131	9.30	1504	This study
MIL-68(Al)I	N.D.	ca. 50–300	N.D.	1386.4	^22^
MIL-68(Al)T-0	82.3	439	4.33	959.8	This study
MIL-68(Al)T-4	87.5	1109	3.40	1077	This study
MIL-68(Al)T-12	82.8	2143	1.56	1384	This study
MIL-68(Al)T-20	46.2	2282	–0.98	1492	This study
MIL-68(Al)T	N.D.	N.D.	N.D.	1372	^22^
MIL-68(Al)T	67	ca. 100	N.D.	1040	^28^
MIL-53(Al)M-0	60.2	67	0.54	860.2	This study
MIL-53(Al)M-4	59.1	99	7.13	934.4	This study
MIL-53(Al)M-12	54.6	100	8.70	1103	This study
MIL-53(Al)M-20	48.9	120	11.63	1257	This study
MIL-53(Al)M	52	178	N.D.	895	^23^
MIL-53(Al)D-0	86.4	77	10.3	1033	This study
MIL-53(Al)D-4	88.7	135	5.70	1113	This study
MIL-53(Al)D-12	95.5	182	5.78	1403	This study
MIL-53(Al)D-20	94.6	191	4.50	1376	This study
MIL-53(Al)D	ca. 100	41	N.D.	1084	^23^
MIL-53(Al)D	N.D.	66.3	N.D.	1160.7	^24^

a N.D. = no data.

### 3.3 Thermal Stability and Missing-Linker Ratio

TG analysis was conducted in air to evaluate the thermal stability and missing-linker ratio in the MOFs. Figure 4 shows the TG curves of the 16 MOFs. In all measurements, weight losses due to the desorption of preadsorbed molecules below 473 K and the combustion of MOFs at high temperatures were observed. In MOFs prepared in pure organic solvents, thermal decomposition and combustion began at lower temperatures. In contrast, the MOFs synthesized using the water-modulation method exhibited high thermal stability. For example, the decomposition and combustion of MIL-68­(Al)­T-0 and MIL-68­(Al)­T-20 began at approximately 550 and 670 K, respectively. The difference in thermal stability under an oxidative atmosphere is based on the change in crystallinity. Bokhoven et al. studied the relationship between the crystallinity and thermal stability of UiO-66, and reported that ligands that were not completely incorporated into the framework were easily attacked under oxidative conditions.^27^

**Figure 4:**
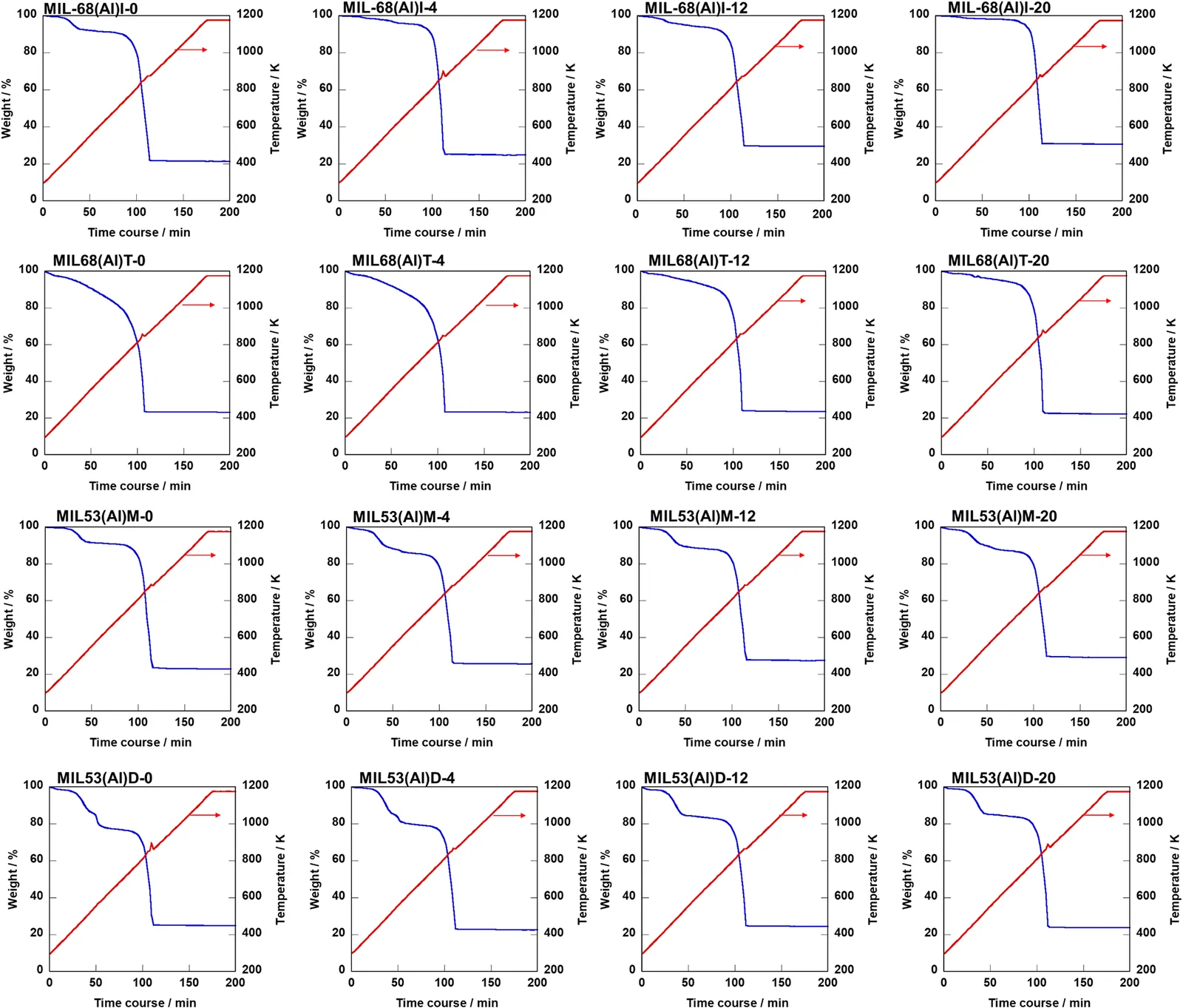
TG curves of the prepared Al-based MOFs. Measurement was conducted with the heating rate of 5 K min^–1^ in air.

Figure 5 provides the relationship between the water content in the solvent and the missing-linker ratio calculated from the TG measurements. In the protonic-solvent systems MIL-68­(Al)I and MIL-53­(Al)­M, the missing-linker ratio increased with the amount of water added. Although the number of missing linkers was very small without the addition of water in both cases, MIL-68­(Al)­I-20 and MIL-53­(Al)­M-20 had the ratios of 9.30% and 11.63%, respectively. This behavior is similar to that reported for CAU-1­(Al)-NH_2_ synthesized in methanol, a protonic solvent. Adding 5 wt % water increased the missing-linker ratio of CAU-1­(Al)-NH_2_ from 0% to 5.4%.^21^ In contrast, water modulation decreased the ratio in aprotic solvents, from 4.3% to −0.98% in MIL-68­(Al)­T and from 10.3% to 4.5% in MIL-53­(Al)­D. It should be noted that slightly negative missing-linker ratios originate from the experimental uncertainty associated with the TG-based estimation and should be regarded as approximately zero.

**Figure 5:**
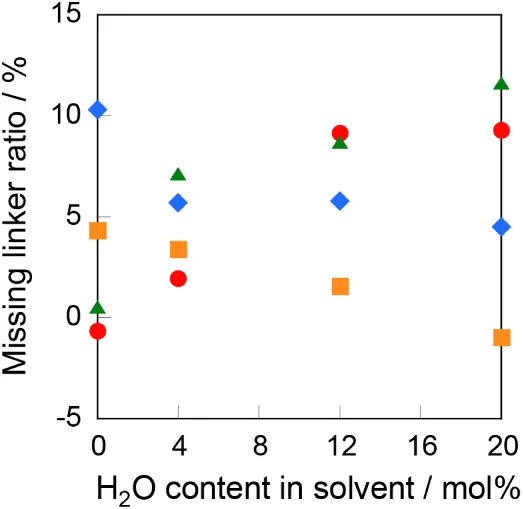
Missing-linker ratios calculated from TG curves. ◦MIL-68­(Al)­I, □MIL-68­(Al)­T, △MIL-53­(Al)­M, ◊MIL-53­(Al)­D.

### 3.4 BET Surface Area

Figure 6 shows the adsorption isotherms of N_2_ at 77 K on the prepared samples, which were measured to evaluate their BET surface areas. Table 2 compares the BET surface areas with literature values.^22–24,28^ The MOFs prepared without the addition of water had BET surface areas comparable to those reported in the literature. In addition, the adsorbed amounts of N_2_ and the BET surface areas of the MOFs increased with the addition of water. For example, BET surface areas of Al-based MOFs improved by approximately 30%–50% with 20 mol % water in the solvent. In all MOFs prepared in this study, BET surface areas drastically increased by this simple method.

**Figure 6:**
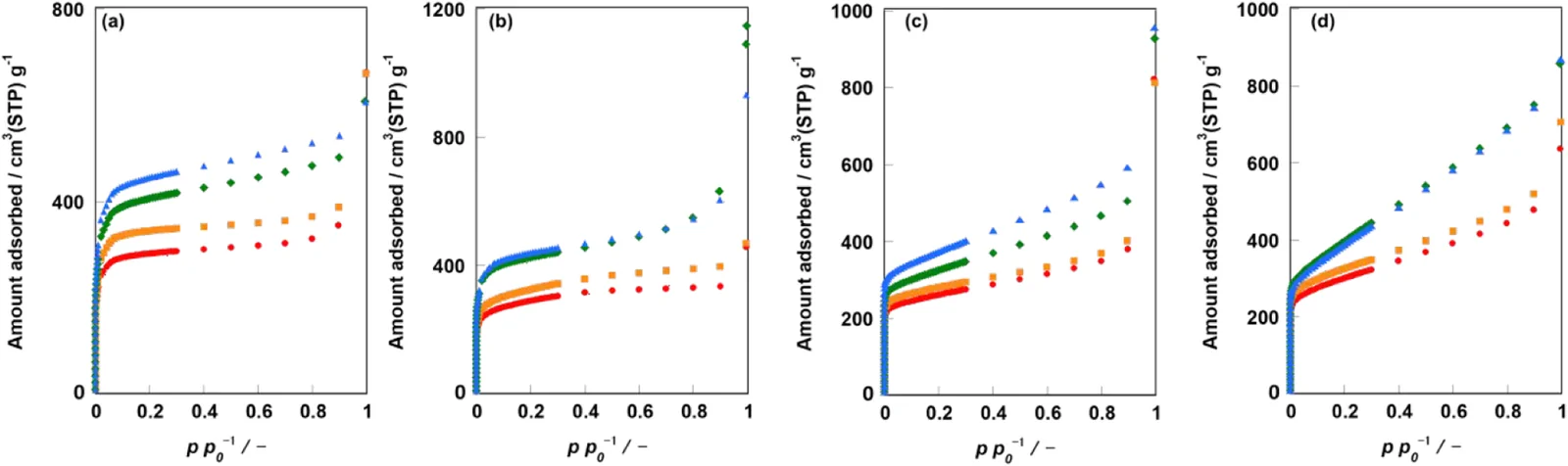
N_2_ adsorption isotherms at 77 K on (a) MIL-68­(Al)­I, (b) MIL-68­(Al)­T, (c) MIL-53­(Al)­M, and (d) MIL-53­(Al)­D. ◦0, □4, ◊12, and △20 mol % water in the solvent.

## 4 Discussion

First, the role of water in the crystallization of Al-based MOFs in protonic solvents is discussed. As described above, monocarboxylic acids are often employed as modulators for controlling crystallization behavior. Among these modulation methods, improvements in the crystal size and BET surface area have been widely reported.^11–14^ The nucleation frequency decreases because of the competitive coordination of the monocarboxylic acid and the original ligand with the metal cation, resulting in a larger crystal size as the number of crystals decreases. As in the case of MIL-68­(Al)I and MIL-53­(Al)­M, water addition decreased the product yield and increased the particle size. The effect of water is similar to that of monocarboxylic acids in conventional modulation syntheses, suggesting that water acts as a modulator that inhibits the coordination between the Al cation and terephthalic acid. These results agree with those of the CAU-1­(Al)-NH_2_ synthesis in methanol with water in the previous study.^21^ Tanaka et al. reported that the formation of coordination bonds between metal-water and metal–ligand occurred competitively in ZIF-8 synthesis using water as the solvent.^29,30^ In their study, the ratio of ligand/metal/water affected the crystal morphology and particle size. When the ligand/metal ratio was small, metal–ligand bonds were less likely to form, resulting in a lower frequency of nucleation and a larger crystal size. They reported that the crystal size increased from 0.3 to 3.4 μm when the 2-methylimidazole/Zn ratio decreased from 100 to 40^30^. This phenomenon also indicated that water interferes with the formation of coordination bonds between the metal and original ligand.

Regardless of the organic solvent species, along with an increase in the mean particle size and decrease in the number of particles, a broadening of the particle size distribution was also observed. In particular, although the yield is almost constant (86–95%) in MIL-53­(Al)­D, the number distribution of particle size changed. Ostwald ripening,^31,32^ in which smaller crystals dissolve and larger crystals subsequently grow, could be one possible explanation for this observation. Such a growth mode possibly occurred because both the dissolution and recrystallization of the MOFs were promoted by the presence of water. However, the present results do not provide direct evidence for this mechanism, and time-resolved crystallization studies would be required.

In the synthesis using aprotic organic solvents, such as THF and DMF, water has a different role. Although water increased the particle size and BET surface area, as in protonic solvents, water decreased the missing-linker ratio. In addition, an appropriate amount of water in aprotic solvents enhanced the product yields, unlike in protonic solvents. The XRD results and product yields indicated that crystallization was promoted by an appropriate amount of water. Although direct experimental evidence is not yet available, I consider that a small amount of water enhances deprotonation of the organic ligand, which is the first step in MOF crystallization. The addition of water to an aprotic solvent facilitates linker deprotonation by increasing the polarity of the solvent and enhancing the stability of the deprotonated species. Studies have reported that the crystallization of MOFs is promoted by aqueous solutions of inorganic acids, such as MIL-101­(Cr) with hydrofluoric acid,^33^ MIL-101­(Cr) with nitric acid,^34^ UiO-66­(Zr) and UiO-67­(Zr) with hydrochloric acid.^35^ Adding small amount of inorganic acid is resembles the experimental conditions in this study, in which water was added to aluminum nitrate or aluminum chloride.

The effect of modulators on the missing-linker content has been widely reported; however, the mechanism remains unclear. In some cases, the addition of a monocarboxylic acid as a modulator increased the number of missing linkers.^36^ For example, Truong et al. reported that the number of missing linkers per Zr_6_ unit in UiO-66­(Zr) increased with the amount of 4-aminobenzoic acid.^36^ On the other hand, it has also been reported that the modulators may have little effect on the number of missing linkers. Janiak et al. synthesized MIL-160­(Al) in water and pointed out that the modulator/linker ratio was important for obtaining a positive effect on the introduction of linker defects or the improvement of crystallinity and crystal size.^20^ In our experiments, the number of missing linkers increased in the protonic organic solvents and decreased in the aprotic organic solvents. In protic solvents, water is presumed to compete with organic linkers for coordination to metal ions, leading to an increase in the number of missing linkers. In contrast, in aprotic solvents, water may promote linker deprotonation, thereby facilitating metal–linker coordination and suppressing the formation of missing linkers owing to insufficient crystal growth. In summary, water plays another role in aprotic solvents to promote crystal growth in addition to acting as a modulator that suppresses the nucleation frequency. This hypothesis can explain the behavior of the product yield in aprotic solvents; for example, the yield reaches a maximum when an appropriate amount of water is added. Figure 7 illustrates the proposed role of water in protonic and aprotic solvents. Further investigations are required to verify this hypothesis.

**Figure 7:**
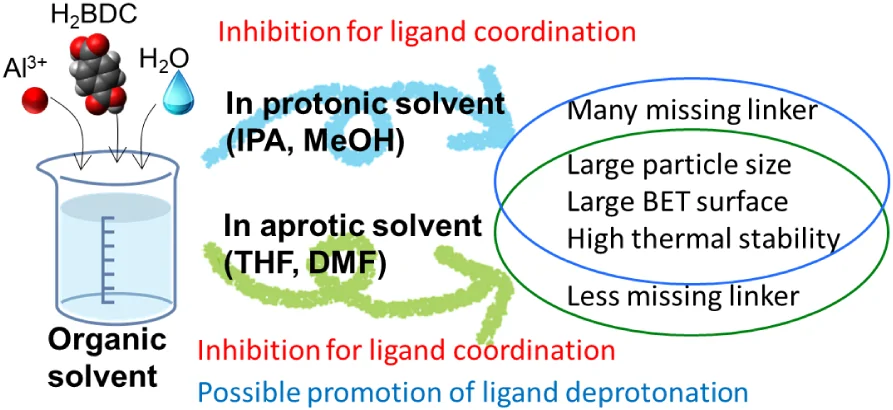
Proposed role of water for MIL-68­(Al) and MIL-53­(Al) synthesis in protonic and aprotic solvents.

## 5 Conclusions

MIL-68­(Al) and MIL-53­(Al) were prepared using a water modulation method. Regardless of the crystal structures and solvent, pure MIL-68­(Al) and MIL-53­(Al) were synthesized. The addition of water increased the particle size and crystallinity. In contrast, the number of crystals decreased, indicating that the water added to the organic solvent suppressed nucleation by competitive coordination of water and ligands with metal ions. Water addition increased the BET surface areas of all MOFs, possibly because the high crystallinity contributed to the large adsorption capacity. This study provides a strategy for controlling the crystallization of Al-MOFs via simple water modulation.

## References

[1] Jia T., Gu Y., Li F. (2022). Progress and potential of metal-organic frameworks
(MOFs) for gas storage and separation: A review. J. Env. Chem. Eng..

[2] Qian Q., Asinger P. A., Lee M. J., Han G., Rodriguez K. M., Lin S., Benedetti F. M., Wu A. X., Chi W. S., Smith Z. P. (2020). MOF-based
membranes for gas separations. Chem. Rev..

[3] Sakai M., Ito A., Matsumoto T., Matsukata M. (2024). One-pot synthesis of HKUST-1 monolith
for CO_2_ adsorption. Chem. Lett..

[4] Konnerth H., Matsagar B. M., Chen S. S., Prechtl M. H. G., Shieh F.-K., Wu K. C.-W. (2020). Metal-organic
framework (MOF)-derived catalysts for
fine chemical production. Coord. Chem. Rev..

[5] Wang Q., Gao Q., Al-Enizi A. M., Nafady A., Ma S. (2020). Recent advances in
MOF-based photocatalysis: Environmental remediation under visible
light. Inorg. Chem. Front..

[6] Lin B.-H., Chuang C.-H., Hsiao L.-W., Wang H.-Y., Chi L.-T., Lin Y.-H., Lin L.-C., Kang D.-Y. (2026). Controlled secondary
growth of CAU-1-NH_2_ membranes with improved CO_2_ separation performance. Langmuir.

[7] Bàez R., Marchini L., Delgado J. A. C., San Martín S., Verdugo F., Araújo K. C., Pereira E. C., Varma R. S., Paixão M. W., Jiménez C. A. (2025). MOF MIL-53­(Al) catalyzed hydroacylation
of azodicarboxylates: Sustainable catalyst for heterogeneous acylhydrazide
synthesis. RSC Adv..

[8] Salimi S., Farnia S. M. F., Akhbari K., Tavasoli A. (2023). Engineered catalyst
based on MIL-68­(Al) with high stability for hydrogenation of carbon
dioxide and carbon monoxide at low temperature. Inorg. Chem..

[9] Sakai M., Hori H., Matsumoto T., Matsukata M. (2023). One-pot synthesis
method of MIL-96 monolith and its CO_2_ adsorption performance. ACS Appl. Mater. Interfaces.

[10] Tanaka S., Fujita K., Miyake Y., Miyamoto M., Hasegawa Y., Makino T., Van der
Perre S., Cousin Saint Remi J.
C. S., Van Assche T., Baron G. V., Denayer J. F. M. (2015). Adsorption and
diffusion phenomena in crystal size engineered ZIF-8 MOF. J. Phys. Chem. C.

[11] Ohsaki S., Morimoto Y., Watanabe S., Tanaka H., Miyahara M. T. (2018). Diffusion
phenomena of propane and propylene in colloidal zeolitic imidazolate
framework-8 particles. J. Taiwan Inst. Chem.
Eng..

[12] Ohsaki S., Watanabe S., Tanaka H., Miyahara M. T. (2017). Free energy analysis
for adsorption-induced structural transition of colloidal zeolitic
imidazolate framework-8 particles. J. Phys.
Chem. C.

[13] Zheng X., Qi S., Cao Y., Shen L., Au C., Jiang L. (2021). Morphology
evolution of acetic acid-modulated MIL-53­(Fe) for efficient selective
oxidation of H_2_S. Chin. J. Catal..

[14] Jiang S., Zhao Z., Chen J., Yang Y., Ding C., Yang Y., Wang Y., Liu N., Wang L., Zhang X. (2022). Recent research progress and challenges of MIL-88­(Fe) from synthesis
to advanced oxidation process. Surf. Interfaces.

[15] Chen Y., Huang X., Zhang S., Li S., Cao S., Pei X., Zhou J., Feng X., Wang B. (2016). Shaping of metal-organic
frameworks: From fluid to shaped bodies and robust foams. J. Am. Chem. Soc..

[16] Bazer-Bachi D., Assié L., Lecocq V., Harbuzaru B., Falk V. (2014). Towards industrial
use of metal-organic framework: Impact of shaping
on the MOF properties. Powder Technol..

[17] Diring S., Furukawa S., Takashima Y., Tsuruoka T., Kitagawa S. (2010). Controlled
multiscale synthesis of porous coordination polymer in nano/micro
regimes. Chem. Mater..

[18] Morris W., Wang S., Cho D., Auyeung E., Li P., Farha O. K., Mirkin C. A. (2017). Role of
modulators in controlling
the colloidal stability and polydispersity of the UiO-66 metal-organic
framework. ACS Appl. Mater. Interfaces.

[19] Canossa S., Gonzàlez-Nelson A., Shupletsov L., Del Carmen Martin M. C., Van der Veen M. A. (2020). Overcoming crystallinity limitations
of aluminium metal-organic frameworks by oxalic acid modulated synthesis. Chem. -Eur. J..

[20] Steinert D. M., Schmitz A., Fetzer M., Seiffert P., Janiak C. (2022). A caveat on
the effect of modulators in the synthesis of the aluminum furandicarboxylate
metal-organic framework MIL-160. Z. Anorg. Allg.
Chem..

[21] Sakai M., Tomita M., Matsukata M. (2026). Water modulated synthesis of highly
crystalline CAU-1-NH_2_. Next Nanotechnol..

[22] So P. B., Tang P.-H., Liao B.-S., Sathishkumar N., Chen H.-T., Lin C.-H. (2021). Sustainable scale-up synthesis of
MIL-68­(Al) using IPA as solvent for acetic acid capture. Microporous Mesoporous Mater..

[23] Warfsmann J., Tokay B., Champness N. R. (2018). Synthesis
of hydrophobic MIL-53­(Al)
nanoparticles in low molecular weight alcohols: Systematic investigation
of solvent effects. CrystEngComm.

[24] Meshram A.
A., Sontakke S. M. (2021). Synthesis
of highly stable nanoscale MIL-53 MOF and
its application for the treatment of complex mixed dye solutions and
real-time dye industry effluent. Sep. Purif.
Technol..

[25] Ahnfeldt T., Guillou N., Gunzelmann D., Margiolaki I., Loiseau T., Férey G., Senker J., Stock N. (2009). [Al_4_(OH)_2_(OCH_3_)_4_(H_2_N-bdc)_3_]·xH_2_O: A 12-connected porous metal-organic
framework with an unprecedented aluminum-containing brick. Angew. Chem., Int. Ed..

[26] Shearer G. G., Chavan S., Bordiga S., Svelle S., Olsbye U., Lillerud K. P. (2016). Defect engineering:
Tuning the porosity and composition
of the metal-organic framework UiO-66 via modulated synthesis. Chem. Mater..

[27] Athar M., Rzepka P., Thoeny D., Ranocchiari M., van Bokhoven J. A. (2021). Thermal degradation of defective
high-surface-area
UiO-66 in different gaseous environments. RSC
Adv..

[28] Seoane B., Sebastiàn V., Téllez C., Coronas J. (2013). Crystallization in
THF: The possibility of one-pot synthesis of mixed matrix membranes
containing MOF MIL-68­(Al). CrystEngComm.

[29] Kida K., Okita M., Fujita K., Tanaka S., Miyake Y. (2013). Formation
of high crystalline ZIF-8 in an aqueous solution. CrystEngComm.

[30] Tanaka S., Kida K., Okita M., Ito Y., Miyake Y. (2012). Size-controlled
synthesis of zeolitic imidazolate framework-8 (ZIF-8) crystals in
an aqueous system at room temperature. Chem.
Lett..

[31] Kwon H. T., Jeong H.-K., Lee A. S., An H. S., Lee T., Jang E., Lee J. S., Choi J. (2016). Defect-induced ripening
of zeolitic-imidazolate framework ZIF-8 and its implication to vapor-phase
membrane synthesis. Chem. Commun..

[32] Wang J., Wang Y., Liu Y., Wu H., Zhao M., Ren Y., Pu Y., Li W., Wang S., Song S., Liang X., He G., Han Y., Jiang Z. (2022). Ultrathin
ZIF-8 membrane through inhibited Ostwald ripening for high-flux C_3_H_6_/C_3_H_8_ separation. Adv. Funct. Mater..

[33] Kim J., Lee Y.-R., Ahn W.-S. (2013). Dry-gel conversion synthesis of Cr-MIL-101
aided by grinding: High surface area and high yield synthesis with
minimum purification. Chem. Commun..

[34] Zhao T., Jeremias F., Boldog I., Nguyen B., Henninger S. K., Janiak C. (2015). High-yield, fluoride-free
and large-scale synthesis
of MIL-101­(Cr). Dalton Trans..

[35] Gökpinar S., Diment T., Janiak C. (2017). Environmentally benign dry-gel conversions
of Zr-based UiO metal-organic frameworks with high yield and the possibility
of solvent re-use. Dalton Trans..

[36] Vo T. K., Kim J., Park J., Dao D. Q., Truong H. B. (2024). Aminobenzoate-defected
UiO-66­(Zr)–NH_2_ frameworks: Scalable synthesis and
characterizations for adsorptive denitrogenation from model fuel. Chem. Eng. J..

